# Supplementary material for: Proteotranscriptomic Profiling of the Toxic Mucus of Kulikovia alborostrata (Pilidiophora, Nemertea)
Source: Toxins (Basel). 2024 Dec 26;17(1):5. doi: 10.3390/toxins17010005 (PMC11768458; doi:10.3390/toxins17010005)
Supplement: Supplementary file 1 [file toxins-17-00005-s001.zip › Supplementary File S4.zip!/ORF 076246 LINALB/5646dc0998f06131/hit_summary.html]

Phyre 2 Results for putative\_toxin\_cistein\_rich\_

|  |  |
| --- | --- |
| Email | vas9i-kz@mail.ru |
| Description | putative\_toxin\_cistein\_rich\_ |
| Date | Wed Apr 5 07:02:50 BST 2023 || Unique Job ID | 5646dc0998f06131 |

|  |  |
| --- | --- |
| Detailed template information |  |

| # | Template | Alignment Coverage | 3D Model | Confidence | % i.d. | Template Information |
| --- | --- | --- | --- | --- | --- | --- |

|  |  |  |  |  |  |  |
| --- | --- | --- | --- | --- | --- | --- |
| 1 | c6enaA\_ | --- \| --- \| --- |  | 94.4 | 52 | **PDB header:**toxin **Chain:** A: **PDB Molecule:**nemertide alpha-1;  **PDBTitle:** nemertide alpha-1  **PDB Entry:** PDBe RCSB PDBj |
| 2 | d1h5oa\_ | --- \| --- \| --- |  | 22.4 | 38 | **Fold:** Defensin-like **Superfamily:** Defensin-like **Family:** Myotoxin **PDB entry:** PDBe RCSB PDBj |
| 3 | c1z99A\_ | --- \| --- \| --- |  | 18.4 | 38 | **PDB header:**toxin **Chain:** A: **PDB Molecule:**crotamine;  **PDBTitle:** solution structure of crotamine, a myotoxin from crotalus durissus2 terrificus  **PDB Entry:** PDBe RCSB PDBj |
| 4 | d1vtxa\_ | --- \| --- \| --- |  | 44.8 | 42 | **Fold:** Knottins (small inhibitors, toxins, lectins) **Superfamily:** omega toxin-like **Family:** Spider toxins **PDB entry:** PDBe RCSB PDBj |
